# Mitochonic Acid-5 Inhibits Reactive Oxygen Species Production and Improves Human Chondrocyte Survival by Upregulating SIRT3-Mediated, Parkin-dependent Mitophagy

**DOI:** 10.3389/fphar.2022.911716

**Published:** 2022-06-06

**Authors:** Ruobing Xin, Yiyang Xu, Dianbo Long, Guping Mao, Hongyi Liao, Ziji Zhang, Yan Kang

**Affiliations:** ^1^ Department of Joint Surgery, The First Affiliated Hospital of Sun Yat-sen University, Guangzhou, China; ^2^ Guangdong Provincial Key Laboratory of Orthopedics and Traumatology, Guangzhou, China; ^3^ Department of Orthopedics, Fujian Provincial Hospital/Shengli Clinical Medical College, Fujian Medical University, Fuzhou, China

**Keywords:** mitochondrial dysfunction, mitochondrial homeostasis, mitophagy, osteoarthritis, pathogenesis, MA-5

## Abstract

Mitochondrial dysfunction is related to the pathogenesis of osteoarthritis (OA); however, there are no effective drugs to treat OA for maintaining mitochondrial homeostasis. Studies have shown that mitochonic acid-5 (MA-5) has a protective effect against mitochondrial damage and plays a role in mitophagy. However, it is not clear whether MA-5 has a beneficial effect on inflammatory articular cartilage. Here, human OA cartilage was obtained from patients undergoing total joint replacement. Interleukin-1β (IL-1β) was used to stimulate chondrocytes and induce inflammatory injury. Cell Counting Kit-8, TUNEL, and flow cytometry assays were used to assess apoptosis. Gene expression was examined using quantitative reverse transcription-polymerase chain reaction. Mitochondrial function was evaluated using immunoblotting, mitochondrial membrane potential assay, JC-1 staining, and immunofluorescence analysis. Mitophagy was detected using immunoblotting and immunofluorescence. 3-(1H-1,2,3-triazol-4-yl) pyridine (3-TYP), a specific inhibitor of Sirtuin 3 (SIRT3), was used to block the SIRT3/Parkin pathway. Mitophagy in the cartilage sections was evaluated via immunohistochemistry. IL-1β was found to induce chondrocyte apoptosis by inhibiting SIRT3 expression and mitophagy. In addition, inflammatory damage reduced the mitochondrial membrane potential and promoted the production of intracellular reactive oxygen species (ROS), leading to increased mitochondrial division, mitochondrial fusion inhibition, and the consequent mitochondrial damage. In contrast, the MA-5 treatment inhibited excessive ROS production by upregulating mitophagy, maintaining the mitochondrial membrane potential, and reducing mitochondrial apoptosis. After chemically blocking SIRT3 with 3-TYP, Parkin-related mitophagy was also inhibited, an effect that was prevented by pretreatment of the chondrocytes with MA-5, thereby suggesting that SIRT3 is upstream of Parkin. Overall, MA-5 was found to enhance the activity of SIRT3, promote Parkin-dependent mitophagy, eliminate depolarized/damaged mitochondria in chondrocytes, and protect cartilage cells. In conclusion, MA-5 inhibits IL-1β-induced oxidative stress and protects chondrocytes by upregulating the SIRT3/Parkin-related autophagy signaling pathway.

## 1 Introduction

Osteoarthritis (OA) incidence is the highest among elderly individuals, and the disease is characterized by the destruction of chondrocyte homeostasis, oxidative stress, cartilage cell death, and subchondral bone remodeling (Ansari et al., 2020). In OA chondrocytes, interleukin (IL)-1β induces the degradation of the cartilage matrix protease and promotes the expression of other inflammatory mediators (Cho et al., 2019). However, there is a lack of drugs effective drugs that can cure OA.

During recent years, research on the relationship between mitochondrial metabolism and OA has made considerable progress. Mitochondria are key organelles involved in energy metabolism, free radical production, and various signaling pathways (Vafai and Mootha, 2012). Mitochondrial dysfunction is thought to be associated with extracellular matrix metabolism, apoptosis, aging, and a range of pathological processes including arthritis (Blanco et al., 2004; Jaiswal et al., 2015). Mitochondrial dysfunction can lead to various diseases in humans, including OA (Blanco et al., 2011). Oxidative stress affects chondrocytes as well as synovial and subchondral bone cells (Li et al., 2012; Lepetsos and Papavassiliou, 2016).

Mitochondria produce reactive oxygen species (ROS) in cells. When the intracellular ROS levels exceed the metabolic level and the cells cannot eliminate excess ROS, oxidative stress occurs, leading to cytotoxicity (Belenguer-Varea et al., 2020). ROS, in turn, can damage mitochondria, cause mitochondrial dysfunction, Mitochondrial dysfunction causes marked downregulation of superoxide dismutase 2 (SOD2) (Kasai et al., 2020), Superoxide dismutase 2 is a major mitochondrial antioxidant protein, and further enhance ROS production. In response to inflammatory stress, mitochondrial dysfunction promotes the release of ILs, chemokines, and other cytotoxic factors (Jin et al., 2018). The cycle between mitochondria and ROS balance leads to continuous oxidative damage, which eventually leads to cell death (Feng et al., 2017). Therefore, maintaining normal ROS levels is essential for the treatment of OA. In addition, numerous studies have shown that mitochondrial dysfunction-mediated OA pathogenesis also includes oxidative stress, chondrocyte apoptosis, cartilage matrix calcification, autophagy, impaired chondrocyte anabolism and growth response, and cytokine-induced inflammatory response Increase (Terkeltaub et al., 2002; Blanco et al., 2004; Lotz and Loeser, 2012; Lopez de Figueroa et al., 2015).

Mitophagy is the cellular response to mitochondrial damage and dysfunction (Xiao et al., 2017), which effectively promotes the removal of damaged or unrepaired mitochondria, thereby maintaining mitochondrial function and inhibiting mitochondrial apoptosis (Zhou et al., 2017). The molecular mechanism underlying mitophagy involves depolarization of the mitochondrial membrane that leads to a decrease in the extramembrane potential and loss of the potential gradient in the mitochondrial outer membrane. Briefly, putative PTEN-induced kinase 1 (PINK1) can recruit Parkin and other factors to the surface of the mitochondria and initiate mitophagy, and the sequestosome 1 (also named p62) factor binds to the autophagy marker, light chain LC3A, to promote the encapsulation of mitochondria by the isolated membrane. When free LC3A is converted to the phosphatidylethanolamine-conjugated LC3B, it adheres to the outer membrane, promoting the formation of autophagosomes, which are subsequently combined with lysosomes to degrade their content (Shin et al., 2019). Parkin-related mitophagy can reduce ROS-induced damage and improve the survival of human chondrocytes (Ansari et al., 2018). It has been reported that increased Parkin-related mitophagy can enhance the antioxidant defense mechanism, thereby preventing the apoptosis of intervertebral endplate chondrocytes induced by oxidative stress (Kang et al., 2020); however, information on this mechanism is limited. Importantly, loss of Parkin function may be directly involved in the pathogenesis of OA (Lopez de Figueroa et al., 2015).

Sirtuin 3 (SIRT3) is a mitochondrial nicotinamide adenine dinucleotide-dependent deacetylase that primarily resides in the mitochondria (Meng et al., 2018). Its activity is regulated by different pathways involved in protein deacetylation to maintain the function and metabolism of mitochondria under stress (Dai et al., 2014). In addition, SIRT3 can improve cartilage resistance to oxidative stress by rescuing acetylation-dependent inhibition of superoxide dismutase (SOD) 2 activity and preventing the progression of early-stage OA. SIRT3 can also enhance the activity of SOD2 in degenerative cartilage and delay the damage of early OA cartilage by enhancing the resistance of chondrocytes to oxidative stress (Fu et al., 2016). Moreover, it regulates mitochondrial ROS through SOD2 to maintain the activity and differentiation of osteoblasts (Gao et al., 2018).

Mitochonic acid-5 (4-(2,4-difluorophenyl)-2-(1H-indole-3-yl)-4-oxobutanoic acid; MA-5) is a mitochondrial homing drug (Suzuki et al., 2015) that regulates mitochondrial energy metabolism by maintaining the mitochondrial membrane potential (Lei et al., 2018). MA-5 also reduces tumor necrosis factor (TNF)-α-mediated neuroinflammation through Parkin-mediated mitophagy and enhances the adenosine monophosphate-activated protein kinase/SIRT3 pathway (Huang et al., 2019). MA-5 has an activating effect on SIRT3 and mitophagy, but the relationship between SIRT3 and autophagy has not been elucidated. A previous study reported that SIRT3 has a protective effect on the homeostasis of mitophagy Feng et al. (2018). Although several studies have reported the beneficial effects of MA-5 on mitochondria, there are no reports on the effect of MA-5 on human OA cartilage. The main purpose of the present study was to explore the protective effect of MA-5 on chondrocytes during inflammation and verify its underlying molecular mechanisms.

## 2 Materials and Methods

### 2.1 Human Cartilage Collection and Treatment

This study adhered to the standards of the Ethics Committee on Human Experimentation at the First Affiliated Hospital of Sun Yat-sen University, China (2021-334) and to the principles of the Declaration of Helsinki (2000). All participants provided informed consent. Human OA cartilage was obtained from patients undergoing total joint replacement (*n* = 8 mean age: 66 years; range: 58–79 years; male: 4, female: 4). OA was diagnosed according to the American College of Rheumatology (Altman et al., 1986). Osteoarticular cartilage was separated under aseptic conditions and cut into 0.3–0.5-mm pieces, which were then washed thrice with phosphate buffered saline (PBS; Gibco, Waltham, MA, United States) containing 1% penicillin (100 IU/ml) and streptomycin (100 μg/ml). The tissue pieces were treated with 0.25% trypsin (Gibco) at 10–15 times the volume of the tissue for 1 h at 37°C. Next, the tissue pieces were treated with 0.02% type II collagenase (Roche, Basel, Switzerland) and Dulbecco’s modified Eagle medium/nutrient mixture F-12 (DMEM/F-12; Gibco) overnight at 37°C, and then filtered using a 70-µm cell strainer (BS-70-XBS; Biosharp OÜ, Tallinn, Estonia). The filtrate was centrifuged at 1,500 rpm for 3 min to collect the cells. The cells were cultured in DMEM/F-12 (Gibco) containing 10% fetal bovine serum (Gibco) and 1% penicillin/streptomycin solution (Gibco) for 3–7 days. IL-1β (Beyotime, Shanghai, China) or 3-(1H-1,2,3-triazol-4-yl)pyridine (3-TYP; MedChemExpress, Shanghai, China) was used to stimulate chondrocytes 24 h. MA-5 was used to pretreat the cells 2 h, which were then used in the subsequent experiments.

### 2.2 Measuring Cell Viability

According to experimental requirements, the cells were treated with IL-1β and/or MA-5. According to the experimental operation manual, the Cell Counting Kit-8 (CCK-8) (Beyotime) or TdT-mediated dUTP Nick-End Labelling (TUNEL) method (Meilunbio, China) was used to assess cell apoptosis. The results were analyzed using a microplate reader (Bioteck, Vicenza, Italy) or fluorescence microscope (Olympus, Tokyo, Japan).

### 2.3 Western Blotting

Extraction and analysis of cellular proteins were performed as previously described (Mao et al., 2017). Briefly, the cell culture medium was discarded, and the cells were washed twice with PBS. RIPA buffer containing phenylmethylsulfonyl fluoride was used to digest the cells, following which the lysate was collected and centrifuged at 14,000 *g* for 10 min at 4°C. Protein concentration in the supernatant was determined using the BCA assay. After electrophoresis, the proteins were transferred onto polyvinylidene difluoride membranes (Millipore, Bedford, MA, United States). The membranes were incubated with primary antibodies overnight. After washing with Tris-buffered saline-Tween 20 buffer, the proteins were incubated with a secondary antibody at room temperature for 1 h, and then an ECL chemiluminescence kit (Millipore) was used for observation. The following antibodies were used: anti-dynamin-related protein 1 (DRP1; 1:1000 dilution; #12957-1-AP), anti-mitofusin 1 (MFN1; 1:500; #13798-1-AP), anti-mitofusin 2 (MFN2; 1:5000; #67487-1-Ig), anti-SIRT3 (1:500; #10099-1-AP), anti-Parkin (1:1,000; #12957-1-AP), anti-glyceraldehyde 3-phosphate dehydrogenase (GAPDH; 1:20,000; #60004-1-Ig), anti-SOD2 (1:2000; #24127-1-AP), and anti-peroxisome proliferator-activated receptor γ coactivator-1α (PGC-1α; 1:5000; #66369-1-Ig) were purchased from Proteintech (Rosemont, IL, United States); and anti-LC3B (1:500; #AF4650) was procured from Affinity Biosciences (Cincinnati, OH, United States). Results were analyzed using ImageJ version 2.1.0/1.53C (https://imagej.nih.gov/ij/index.html).

### 2.4 RNA Extraction and Real-Time Quantitative Polymerase Chain Reaction (RT-qPCR)

RNA extraction and reverse transcription were performed as previously described (Mao et al., 2017). The following primer sequences were used: *SIRT3* forward, 5′-CCC​CAA​GCC​CTT​TTT​CAC​TTT-3′ and reverse, 5′-CGA​CAC​TCT​CTC​AAG​CCC​A-3′; *GAPDH* forward, 5′-ACA​ACT​TTG​GTA​TCG​TGG​AAG​G-3′ and reverse, 5′-GCC​ATC​ACG​CCA​CAG​TTT​C-3′. The 2^−ΔΔCt^ method was used to calculate relative gene expression.

### 2.5 ROS Detection

After collecting the cells, they were suspended in diluted 2′,7′-dichlorofluorescin diacetate (DCFH-DA) and incubated in a 37°C cell incubator for 20 min. The contents were mixed upside down every 3–5 min to allow maximum contact between the probe and cells. Later, the cells were washed twice with serum-free cell culture medium to remove the excess DCFH-DA. A fluorescence microscope (Olympus) was used to observe the cells, or the cells were analyzed using a flow cytometer (Beckman Coulter, Brea, CA, United States).

### 2.6 Mitochondrial Membrane Potential

Mitochondrial membrane potential was analyzed using JC-1 staining. Chondrocytes were incubated with MA-5 and/or IL-1β for 24 h, after which the cells were washed once with PBS, mixed thoroughly with JC-1 staining working solution (#C2006; Beyotime), and incubated for 20 min at 37°C in a cell incubator. The cells were then washed twice with JC-1 staining buffer. Subsequently, 2 ml of cell culture medium was added to the cells, and they were observed under a fluorescence microscope (Olympus). Normal mitochondria when stained with JC-1 appeared red, whereas damaged mitochondria appeared green. Changes in membrane potential were evaluated by calculating the ratio of red to green fluorescence.

### 2.7 Immunofluorescence Staining

Immunofluorescence was performed as described in a previous study (Haseeb et al., 2017). The cells were fixed with 4% paraformaldehyde for 15 min and washed with PBS thrice for 3 min. The cells were then permeated with 0.5% Triton X-100 at room temperature for 20 min, washed with PBS three times, and blocked using 5% goat serum for 30 min at room temperature (MB4508; Dalian Meilunbio Co., Dalian, China). The cells were then incubated with primary antibodies overnight at 4°C. Later, the cells were washed with PBS three times and incubated with fluorescent secondary antibody (#A0423; Beyotime) at 37°C for 1 h. Thereafter, the cells were washed with PBS three times and stained with 4′,6-diamidino-2-phenylindole (DAPI) for 5 min. Excess DAPI was removed using PBS. MitoTracker Red CMXRos (#C1035-50; Beyotime) was used for staining mitochondria. The cells were observed, and images were collected using a confocal laser microscope (LSM 780; Zeiss, Oberkochen, Germany).

### 2.8 Flow Cytometry

The cells were seeded in a six-well plate. Different treatments were performed after the cells reached approximately 80% confluency. The cells were collected, and then treated using the Annexin V-FITC/PI Apoptosis Detection Kit (Dalian Meilunbio Co.), DCFH-DA (Beyotime), and JC-1 Assay Kit (Beyotime) (Song et al., 2018). Flow cytometry (Beckman Coulter) was used to detect changes in cell apoptosis, ROS levels, and membrane potential.

### 2.9 Immunohistochemical Analysis

OA cartilage specimens were obtained from 6 patients (3 males and 3 females) with an average age of 76 years 4 cases of normal cartilage specimens (3 males and 1 female), with an average age of 32 years, did not suffer from OA or rheumatoid arthritis. Patients who underwent lower limb amputation due to trauma or malignant bone tumor and did not invade the knee joint were treated with These served as the control group. Tissue sections were deparaffinized and washed with PBS thrice after hydration. For antigen retrieval, the sections were treated with citric acid, boiled in a pressure cooker for 5–10 min, and then placed in 3% H_2_O_2_ for 10 min. Next, the sections were washed three times with PBS. The sections were then blocked with 5% goat serum (MB4508; Dalian Meilunbio Co.) at room temperature for 30 min followed by incubation with Parkin, LC3B, P62, and SIRT3 primary antibodies in a 4°C humid chamber overnight. On the next day, the sections were rewarmed at 37°C for 1 h, washed with PBS three times, and incubated with secondary antibodies at room temperature for 1 h. The sections were allowed to dry overnight and then observed under a microscope. Results were analyzed using ImageJ version 2.1.0/1.53C.

### 2.10 Statistical Analyses

Results are expressed as mean ± standard deviation. A one-way analysis of variance or Student’s *t*-test was used to determine the differences between groups. Results with *p* < 0.05 were considered statistically significant. All experiments were performed with three independent biological replicates. SPSS version 26 (IBM Corp., Armonk, NY, United States) was used to perform the statistical analyses.

## 3 Results

### 3.1 MA-5 Protects Chondrocytes From IL-1β-Mediated Negative Effects

**FIGURE 1:**
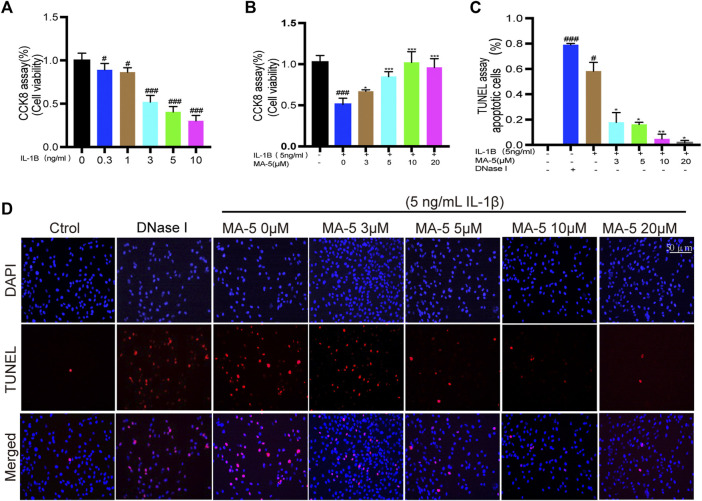
MA-5 protects OA cells from IL-1β-induced apoptosis. **(A,B)** Cell viability was analyzed using the CKK-8 assay. **(C,D)** Apoptosis of OA cells was determined using the TUNEL assay. All data are presented as mean ± standard deviation. ***p < 0.001, **p < 0.01, and *p < 0.05 compared with the IL-1β treatment group; ^#^ *p* < 0.05 and ^###^ *p* < 0.001 compared with the control group, as determined using a one-way analysis of variance.

IL-1β treatment induced inflammation in chondrocytes and maintained cell viability as detected using the CCK-8 assay. As the IL-1β concentration increased, the chondrocyte activity gradually decreased (Figure 1A). The inhibitory effect of 5 ng/ml IL-1β on the cells reached more than 50%, and this concentration was used in the subsequent experiments. Next, chondrocytes were pretreated with different concentrations of MA-5, after which they were treated with IL-1β. It is noteworthy that the inhibitory effect of IL-1β on chondrocytes was blocked by MA-5 (Figure 1B), with increasing concentrations of MA-5 preventing cell apoptosis as determined using TUNEL staining (Figures 1C,D). These results demonstrate the protective effect of MA-5 on chondrocytes during inflammation.

### 3.2 MA-5 Prevents IL-1β-Induced Inhibition of SIRT3 Expression in OA Chondrocytes

**FIGURE 2:**
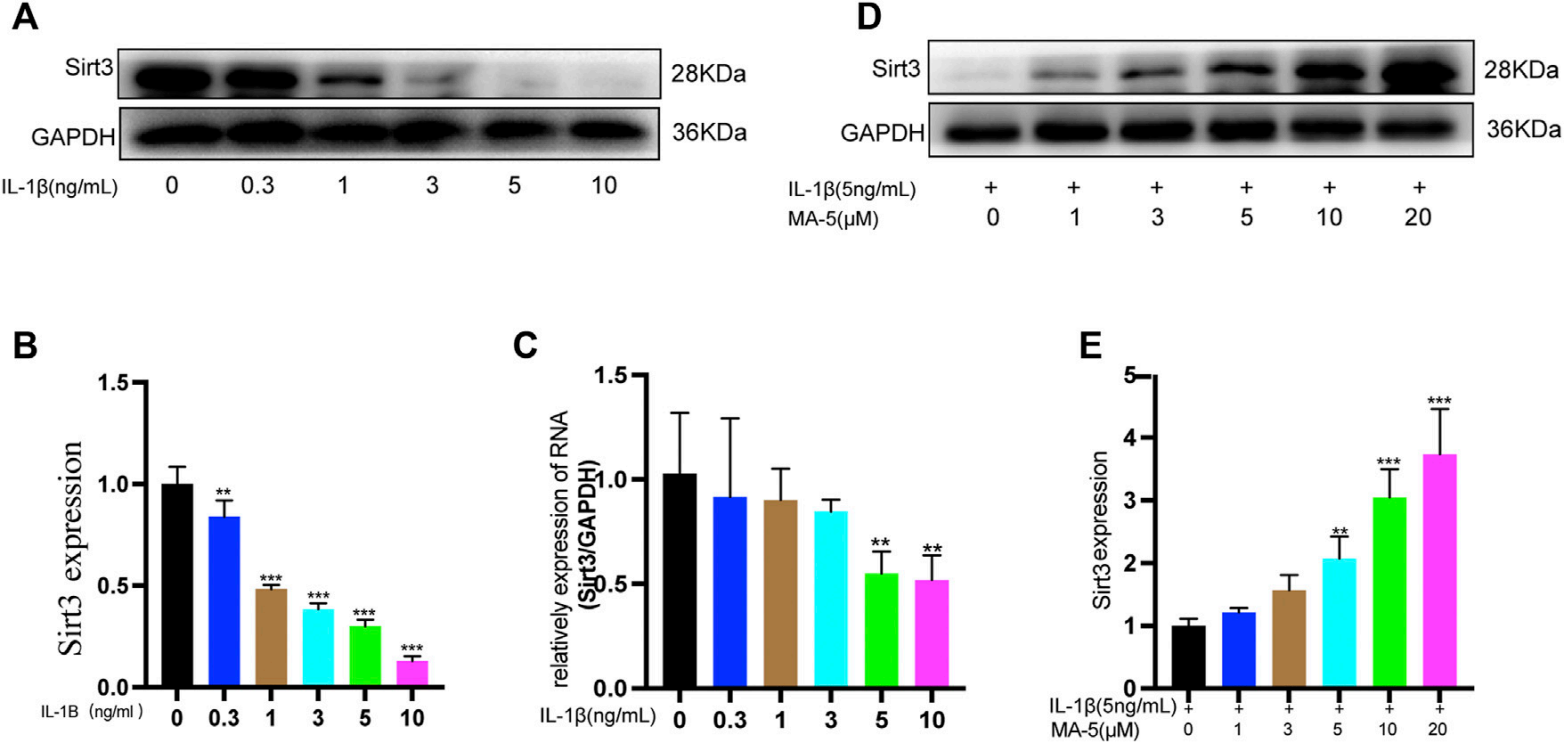
MA-5 prevents the inhibitory effect of IL-1β on SIRT3 in OA cells. **(A,B)** Different concentrations of IL-1β were used to treat OA cells. Western blotting was used to detect SIRT3. **(C)** Different concentrations of IL-1β were used to treat OA cells, and SIRT3 was detected using RT-qPCR. **(D,E)** Cells were pre-treated with different concentrations of MA-5, and then incubated with 5 ng/ml IL-1β. After 24 h, SIRT3 level was determined using western blotting. All data are presented as mean ± standard deviation. **p < 0.01 and *p < 0.05 compared with the control group, as determined using a one-way analysis of variance.

SIRT3 protects mitochondria by regulating protein deacetylation (Ansari et al., 2017). Different concentrations of IL-1β were used to treat chondrocytes, and western blotting was used to detect the expression of SIRT3 in the cells. As the concentration of IL-1β increased, the expression of SIRT3 showed a decreasing trend (Figures 2A,B), a finding that was further validated using RT-qPCR (Figure 2C). Next, SIRT3 expression was evaluated in OA cells upon treatment with different concentrations of MA-5. Overall, SIRT3 expression showed an increasing trend in the presence of higher levels of MA-5 (Figures 2D,E), reaching significantly higher levels at 10 μM MA-5. Therefore, 10 μM MA-5 was used in the follow-up experiments. These results suggest that MA-5 may activate SIRT3 in OA cells.

### 3.3 MA-5 Exerts a Protective Effect on Mitochondria in OA Chondrocytes

**FIGURE 3:**
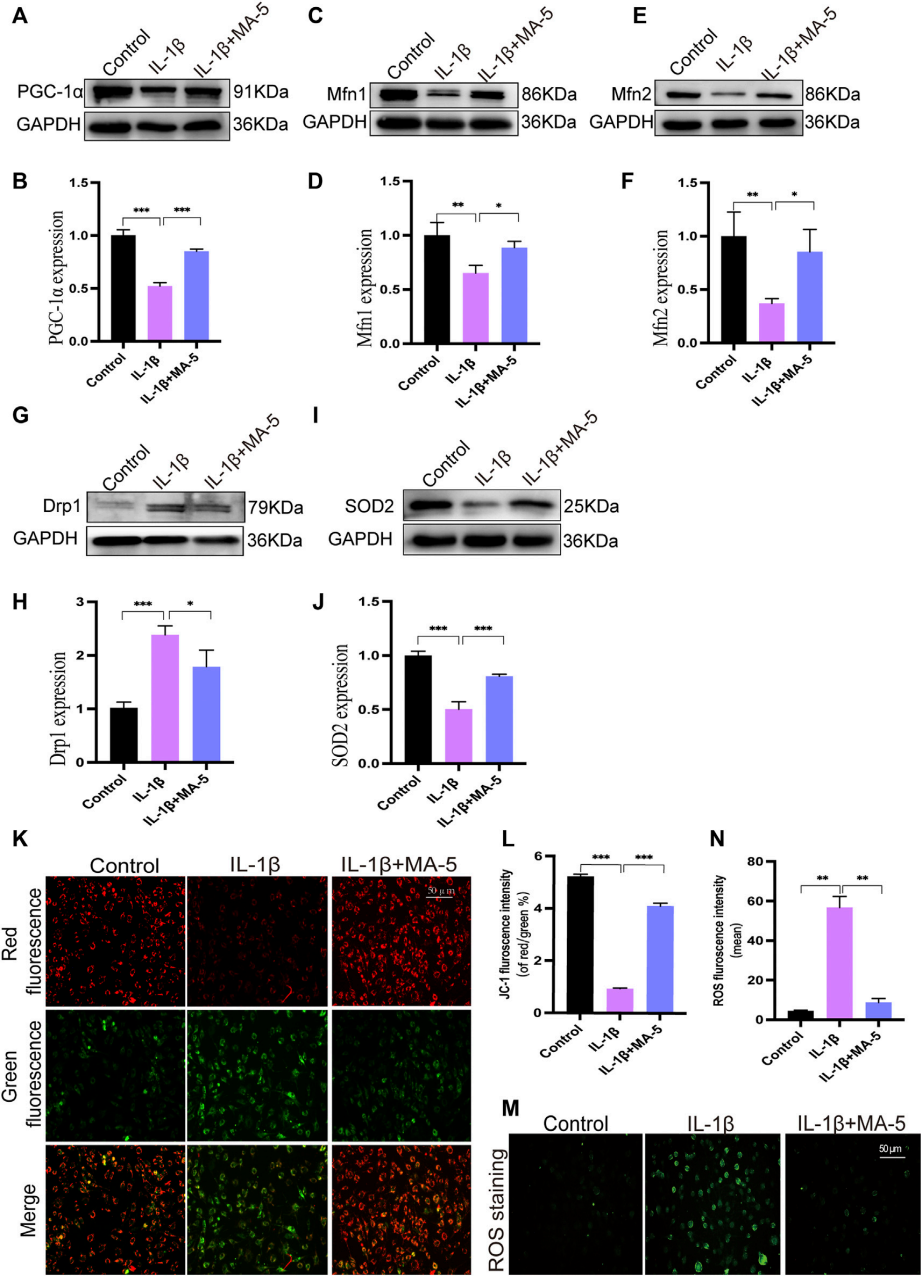
Regulatory effect of MA-5 on mitochondrial function in OA cells. **(A–H)** PGC-1α, MFN1/2, and DRP1 were detected using western blotting. **(I,J)** Changes in SOD2 expression were detected using western blotting. **(K,L)** After JC-1 staining, changes in mitochondrial membrane potential were assessed using a fluorescence microscope. **(M,N)** ROS production was determined using a fluorescence probe. All data are presented as mean ± standard deviation. ***p < 0.001, **p < 0.01, and *p < 0.05 compared with the control group, as determined using a one-way analysis of variance.

We screened and tested mitochondrial fission- and fusion-related proteins, including PGC-1α, MFN1/2, and DRP1 levels, in OA chondrocytes. IL-1β reduced the levels of PGC-1α and MFN1/2, whereas it increased the expression of DRP1. In agreement with the previous results, MA-5 treatment protected the cells from these outcomes (Figures 3A–H).

The membrane potential is an important manifestation of mitochondrial function and is involved in ATP production. JC-1 staining, which enables to assess mitochondrial membrane status, showed that IL-1β treatment reduced the mitochondrial membrane potential, as the amount of green fluorescence signal increased, the ratio of red to green fluorescence signal decreased (Figures 3K,L). In contrast, the mitochondrial membrane potential increased significantly after MA-5 treatment (Figures 3K,L).

High levels of ROS induce mitochondrial dysfunction, which ultimately leads to DNA damage and cell death (Wang and Youle, 2009). Fluorescence microscopy showed that IL-1β treatment resulted in increased intracellular ROS (as determined using the green fluorescence signal), indicating that ROS production increased; an event that was prevented by MA-5 pretreatment (Figures 3M,N). Moreover, IL-1β had an inhibitory effect on the expression of the antioxidant enzyme SOD2, whereas MA-5 promoted its expression (Figures 3I,J). These results suggest that MA-5 inhibits mitochondrial division at the molecular level, maintains mitochondrial membrane potential, and inhibits ROS generation, thereby protecting mitochondria from the negative effects of inflammation.

### 3.4 MA-5 Promotes SIRT3-and Parkin-Associated Mitochondrial Autophagy

**FIGURE 4:**
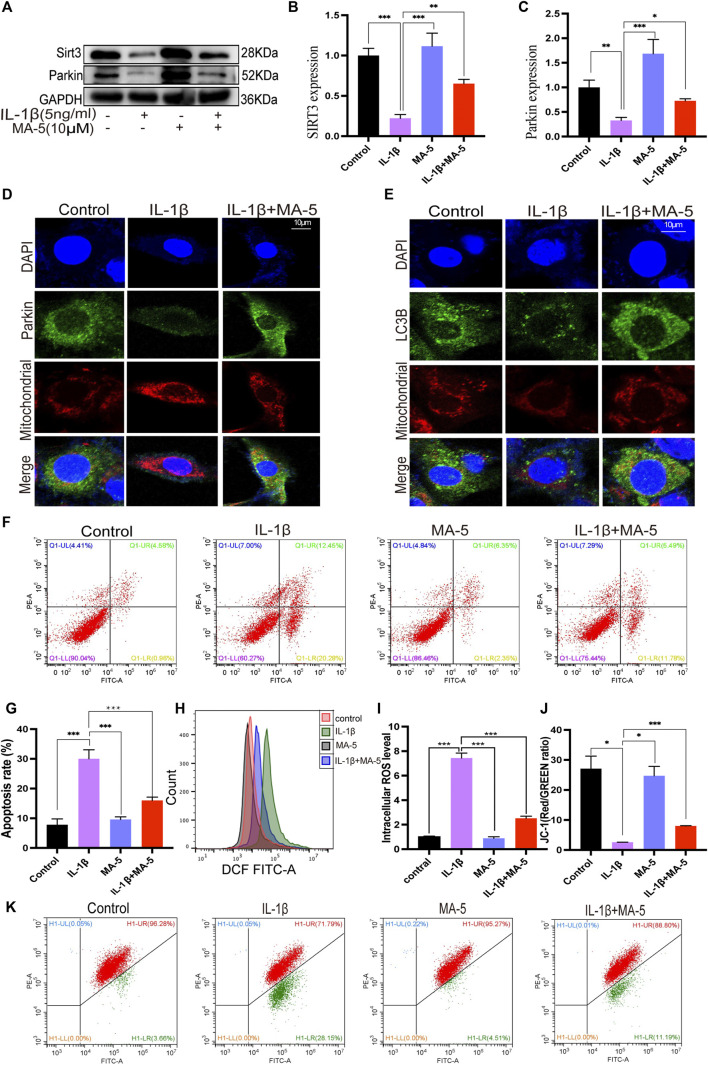
MA-5 promotes SIRT3 expression and activates Parkin-dependent mitophagy. **(A–C)** SIRT3 and Parkin levels in OA cells were detected using western blotting. **(D)** Difference in Parkin fluorescent signal in mitochondria were assessed using confocal microscopy. **(E)** Differences in LC3B expression among different treatment groups were detected via immunofluorescence. **(F–K)** Cell apoptosis, ROS, and membrane potential were detected via flow cytometry. All data are presented as mean ± standard deviation. ***p < 0.001, **p < 0.01, and *p < 0.05 represent differences between groups, as determined using a one-way analysis of variance.(a), (b).

Western blotting was used to clarify the effect of MA-5 on mitophagy and its relationship with SIRT3 and Parkin in chondrocytes. The cells treated with IL-1β showed significantly reduced SIRT3 and Parkin expression. In contrast, MA-5 pretreatment prevented the inhibitory effects of IL-1β on chondrocytes (Figures 4A–C). Moreover, using confocal microscopy, Parkin was found to be significantly reduced in cells treated with IL-1β, an effect that was prevented by MA-5 treatment (Figure 4D). Analysis of the fluorescence signal of LC3B (LC3B is a signal of autophagy) showed an expression and localization pattern that was consistent with the pattern of Parkin (Figure 4E).

The flow cytometry analysis of cell apoptosis rate revealed that IL-1β significantly promotes cell apoptosis, as shown in Figure 4F; the cell apoptosis rate was 32.73%, The apoptosis rate of the cells after MA-5 treatment decreased to 17.27%, indicating that MA-5 has a protective effect on chondrocytes (Figures 4F,G). In addition, on the superimposed image of ROS detected using a flow cytometer, the ROS in the cells treated with IL-1β shifted to the right, indicating that the ROS levels increased, whereas the ROS in the MA-5 treatment group shifted to the left. MA-5 significantly reduced the production of intracellular ROS (Figures 4H,I). The test results of cell membrane potential showed that the ratio of red and green fluorescence in the IL-1β group was lower than that in the MA-5 treatment group, indicating that MA-5 weakened the inhibitory effect of IL-1β on the mitochondrial membrane potential and has a protective effect on mitochondria (Figures 4J,K). Taken together, these results suggest that MA-5 has a common enhancing effect on SIRT3-and Parkin-dependent mitophagy signaling pathways, potentially by inhibiting ROS production.

### 3.5 MA-5 Reverses SIRT3 Inhibitor-Mediated Blockade of the SIRT3/Parkin Signaling Pathway and Its Downstream Effects

**FIGURE 5:**
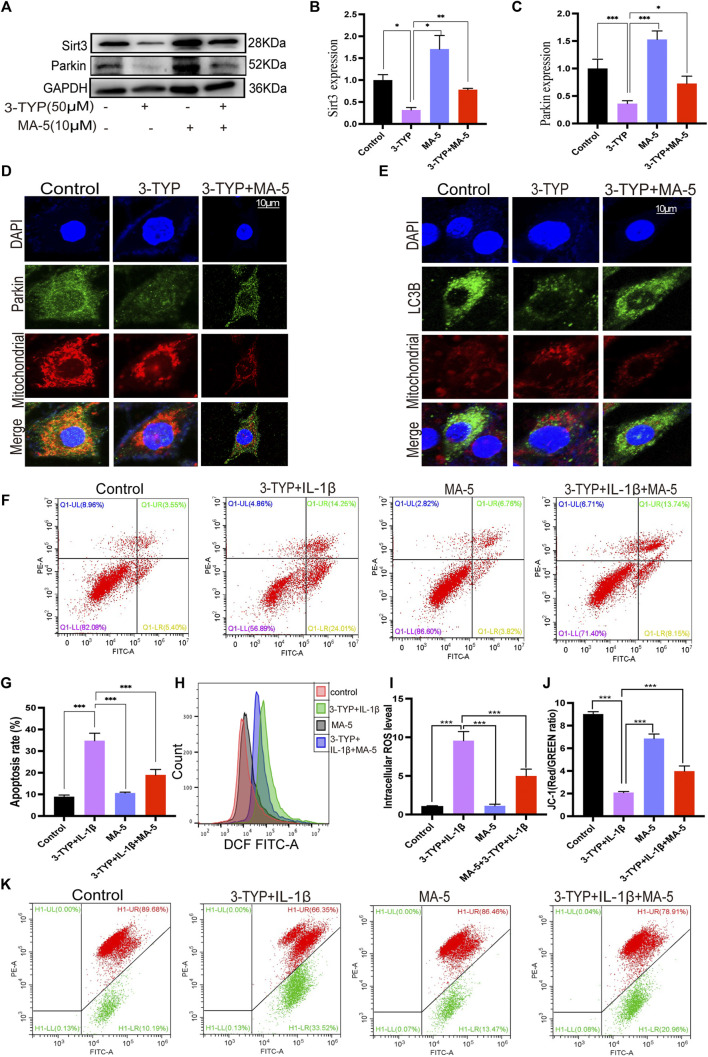
MA-5 prevents the inhibitory effects of 3-TYP on the SIRT3/Parkin axis in OA cells. **(A–C)** Expression of SIRT3 and Parkin were detected using western blotting. **(D)** Difference in Parkin fluorescent signal in mitochondria were assessed using confocal microscopy. **(E)** Differences in LC3B expression were detected via immunofluorescence. **(F–K)** Cell apoptosis, ROS, and membrane potential were detected via flow cytometry. All data are presented as mean ± standard deviation. ***p < 0.001, **p < 0.01, and *p < 0.05 represent differences between groups, as determined using a one-way analysis of variance.

To verify the regulatory relationship between SIRT3 and Parkin in OA, we used the selective SIRT3 inhibitor 3-TYP. The treatment of chondrocytes with 3-TYP reduced SIRT3 and Parkin expression, an effect that was ameliorated by MA-5 pretreatment (Figures 5A–C). These results indicate that SIRT3 exerts a regulatory effect on Parkin, potentially acting as an upstream regulator of the Parkin pathway. Similarly, confocal microscopy showed that the green fluorescence signal of Parkin in chondrocytes treated with 3-TYP was significantly reduced. Moreover, the colocalization of the magenta fluorescence signal decreased, which indicated that the expression of Parkin and LC3B significantly decreased after SIRT3 inhibition. In contrast, pretreatment with MA-5 resulted in increased levels of the fluorescence signals and colocalization of Parkin and LC3B (Figures 5D,E).

The flow cytometric analysis revealed that MA-5 significantly reduced the apoptotic rate of chondrocytes compared with 3-TYP and IL-1β (Figures 5F,G). Additionally, MA-5 significantly reduced the cellular production of ROS (Figures 4H,I) and enhanced the mitochondrial membrane potential (Figures 5J,K). These results further suggest that MA-5 inhibits the production of ROS through the SIRT3/Parkin signaling pathway, thereby protecting mitochondria and chondrocytes.

### 3.6 Impaired SIRT3/Parkin Signals May Contribute for OA Pathogenesis

**FIGURE 6:**
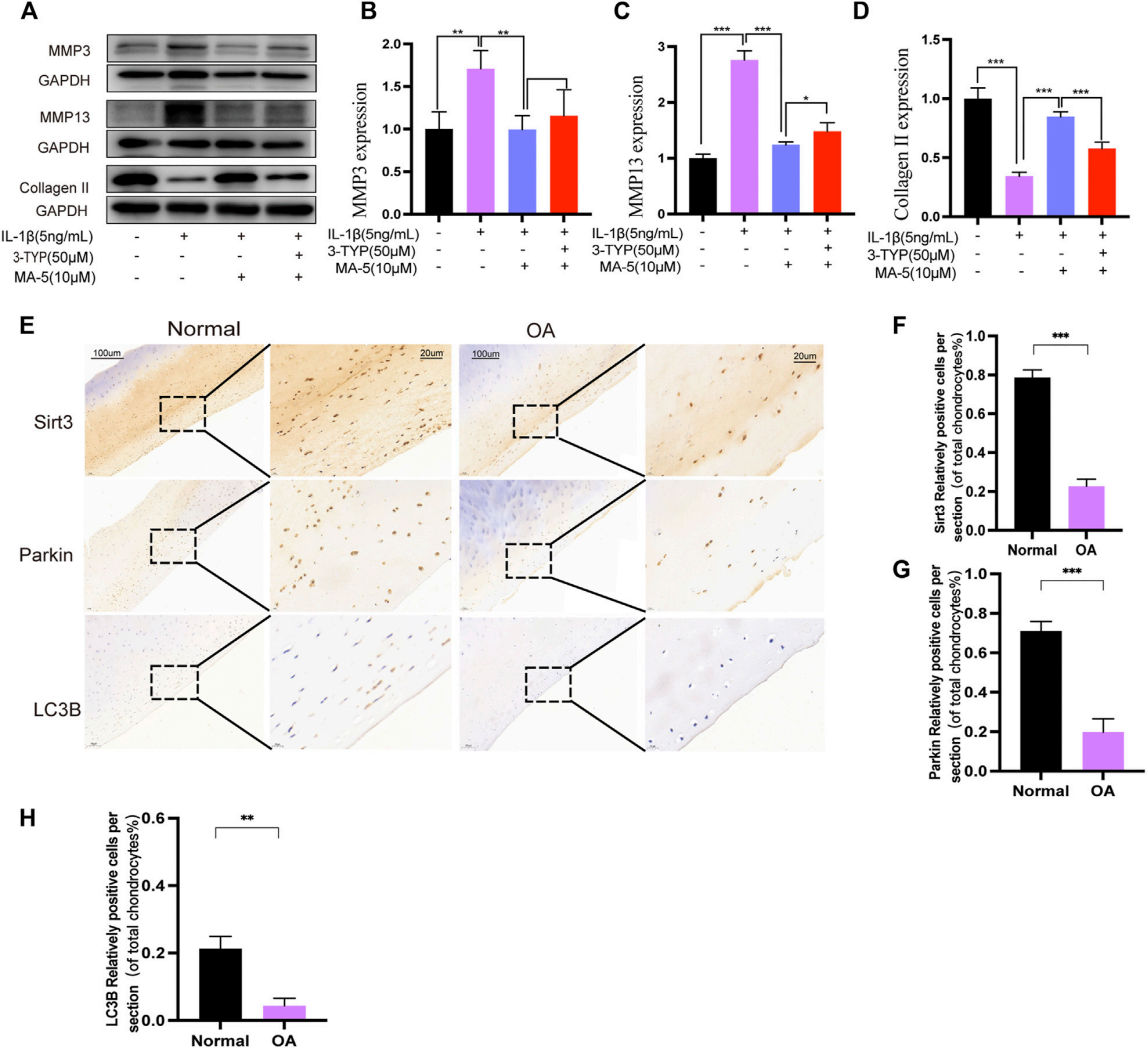
MA-5 inhibited the expression of MMP3 and MMP13 and promoted Collagen II. Number of SIRT3-, Parkin-, and LC3B-positive cells in cartilage samples from patients with OA was significantly lower than that in healthy individuals. **(A–D)** Expression of MMP3, MMP13 and Collagen II were detected using western blotting. **(E)** The number of SIRT3-, Parkin-, and LC3B-positive cells in articular cartilage sections between OA patients and healthy individuals was determined using immunohistochemistry. **(F–H)** Relative ratios of SIRT3-, Parkin-, and LC3B-positive cells in cartilage sections were quantitatively analyzed using ImageJ software. All data are presented as mean ± standard deviation. ***p < 0.001, **p < 0.01, and *p < 0.05 represent differences between groups, as determined using a one-way analysis of variance.

To confirm the protective effect of sirt3-mediated mitophagy on chondrocytes, we examined the expression of OA markers MMP3, MMP13, and Collagen II in IL-1β-treated chondrocytes. As shown (Figures 6A–D), western blotting results showed that the inflammatory cytokine IL-1β increased the levels of matrix-degrading enzymes MMP3 and MMP13 and decreased the level of collagen II in chondrocytes compared with the control group. However, MA-5 treatment reversed the IL-1β-induced inflammatory response, which in turn was attenuated by 3-TYP, an inhibitor of SIRT3. These results suggest that inhibition of SIRT3 enhances the pathological phenotype of chondrocytes through Parkin-dependent mitophagy. Immunohistochemical analysis of SIRT3, Parkin, and LC3B levels in cartilage sections was also performed. The number of SIRT3-, Parkin-, and LC3B-positive cells in the cartilage of patients with OA significantly decreased compared with that in healthy individuals (Figures 6E–H), which suggests that these proteins are necessary to maintain normal cartilage function.

## 4 Discussion

Chondrocyte aging and apoptosis, extracellular matrix degradation with synovial inflammation, and subchondral bone dysfunction are the main pathological changes associated with OA (Malemud, 2017). Nonetheless, there is still no effective treatment for OA, and end-stage OA is still mainly treated surgically. Recently, it was reported that the mitochondrial-homing drug MA-5 not only increases cellular ATP but also protects fibroblasts from patients with mitochondrial disease from cell death (Suzuki et al., 2015; Suzuki et al., 2016). Although MA-5 is expected to become a new drug for treating OA, to our knowledge, there is no report on its effects in this clinical setting. Overall, our study is the first to demonstrate the protective effect of MA-5 on chondrocytes *in vitro*.

IL-1β is a proinflammatory cytokine that is released in inflamed joints by activated synovial cells, chondrocytes, and invading macrophages (Vincenti and Brinckerhoff, 2001). IL-1β can cause the deletion and functional damage of genes essential for stress granule assembly and thus promote OA progression (Ansari and Haqqi, 2016). Therefore, IL-1β is a major inflammatory factor contributing for the development of OA. This is also the reason IL-1β was used in the present study to simulate pathological conditions and establish an isolated OA model. Recent studies have shown that autophagy activity is related to aging; in turn, aging leads to decreased autophagy activity, which eventually promotes chondrocyte injury and apoptosis, thereby leading to osteoarthritis. Autophagy deficiency leads to obvious mitochondrial dysfunction, which is also a pathogenic feature of OA (Caramés et al., 2015; Lopez de Figueroa et al., 2015; Li et al., 2017). Our results are consistent with these previous findings, as they showed that inhibition of autophagy can cause mitochondrial dysfunction and catabolism imbalance, resulting in chondrocyte apoptosis. Indeed, these results demonstrate the protective effect of autophagy on chondrocytes.

Mitochondrial dysfunction is closely related to OA, in which inflammation leads to decreased autophagy, impaired anabolism, and apoptosis of chondrocytes (Blanco and Rego-Perez, 2018). Therefore, inhibition of ROS and inflammation is key to protecting chondrocytes from damage. Treatment with MA-5 showed that it could prevent these inflammation-related damaging effects proving that MA-5 could reduce IL-1β-induced damage to mitochondria at the molecular level and delay the progression of OA. MA-5 can promote the oligomerization of mitochondrial synthase, reduce mitochondrial fragmentation, and restore the structure of the cristae and mitochondrial power, thereby protecting cells from mitochondrial-derived oxidative stress damage (Matsuhashi et al., 2017). Moreover, MA-5 has also been shown to reduce the levels of oxidative stress in TNF-α-induced neuronal inflammation (Huang et al., 2019). In the present study, a similar phenomenon was observed. MFN1 and MFN2 are crucial regulators of mitochondrial fusion in mammalian cells (Wang et al., 2018). PGC-1α is an important stimulating factor in the mitochondrial electron transport chain, and it plays an important role in regulating the generation of ROS (Tran et al., 2011). DRP1 is an essential protein involved in mitochondrial division (Zhang et al., 2021). Their dynamic balance is key to maintaining mitochondrial membrane potential and function. By studying the levels of these factors, we determined the beneficial effects of MA-5 on mitochondria. Noteworthily, the mechanism of this protective effect is still related to ROS inhibition.

We observed an increase in ROS production after IL-1β treatment, which is consistent with the results of previous studies reporting that IL-1β promotes ROS production in chondrocytes *in vivo* (Rojansky et al., 2016). We also observed a decrease in manganese SOD expression, which is present in mitochondria, where it catalyzes the decomposition of superoxide radicals into oxygen and hydrogen peroxide, thereby protecting cells from oxidative damage. In the present study, MA-5 treatment prevented inflammation-related ROS production, further clarifying the beneficial effect of MA-5 on mitochondria.

Mitophagy removes damaged and dysfunctional mitochondria, regulates mitochondrial quality control, and protects mitochondrial function and homeostasis (Eiyama and Okamoto, 2015). PINK1/Parkin is currently the most studied mitophagy pathway, and it has been demonstrated to play a prominent role in the activation of mitochondrial autophagy in nucleus pulposus cells and chondrocytes (Sun et al., 2020). In this study, we also confirmed this finding and that MA-5 promotes Parkin-related mitophagy, which protects chondrocytes and mitochondria from inflammation-promoted damage. By inhibiting SIRT3 expression, we proved that SIRT3 is upstream of Parkin and has a regulatory effect on Parkin expression. Thus, these findings provide new directions for future studies on the treatment of osteoarthritis.

Immunohistochemistry of cartilage samples from healthy individuals and patients with OA further confirmed our hypothesis. SIRT3, Parkin, and LC3B levels were significantly higher in the articular cartilage of healthy individuals than in patients with OA. These results suggest that SIRT3 downregulation and impaired Parkin-dependent autophagy are likely involved in OA pathogenesis. Furthermore, it confirms the protective effect of the Sirt3/Parkin pathway on chondrocytes.

## 5 Conclusion

In this study, we explored for the first time the underlying effects and molecular mechanisms of action of MA-5 on OA cells. MA-5 was found to activate Parkin-dependent mitochondrial autophagy and clear dysfunctional mitochondria by upregulating SIRT3 expression. In addition, MA-5 inhibited the production of ROS through the regulation of the SIRT3/Parkin signaling pathway and improved the survival of chondrocytes during inflammation. Our results revealed potential pathogenic molecular factors; the study paves way for new research for the development of treatment strategies for OA. However, these findings were mainly based on *in vitro* investigations at the cellular level; thus, further *in vivo* studies are warranted.

## Data Availability

The original contributions presented in the study are included in the article/Supplementary Material, further inquiries can be directed to the corresponding authors.

## Abbreviations

AO, Osteoarthritis; CCK-8, Cell Counting Kit-8; DCFH-DA, 2′-7’dichlorofluorescin diacetate; DMEM/F-12, Dulbecco’s Modified Eagle Medium/Nutrient Mixture F-12; DRP1, dynamin-related protein 1; GAPDH, glyceraldehyde 3-phosphate dehydrogenase; IL, interleukin; LC, autophagy marker light chain; MA-5, Mitochonic acid-5; MFN, mitofusin; PBS, phosphate buffered saline; PGC-1α, peroxisome proliferator-activated receptor γ coactivator‐1α; PINK1, PTEN-induced kinase 1; ROS, reactive oxygen species; SIRT3, Sirtuin 3; 3-TYP, 3-(1H-1,2,3-triazol-4-yl)pyridine; TNF, tumor necrosis factor; TVUNEL, TdT-mediated dUTP Nick-End Labelling.
